# Airway Occlusion Pressure and P0.1 to Estimate Inspiratory Effort and Respiratory Drive in Ventilated Children

**DOI:** 10.1097/PCC.0000000000003697

**Published:** 2025-02-13

**Authors:** Michelle W. Rudolph, Maaike Sietses, Alette A. Koopman, Robert G.T. Blokpoel, Martin C.J. Kneyber

**Affiliations:** 1 Department of Paediatrics, Division of Paediatric Critical Care Medicine, Beatrix Children’s Hospital, University Medical Center Groningen, University of Groningen, Groningen, The Netherlands.; 2 Critical Care, Anaesthesiology, Peri-operative and Emergency Medicine (CAPE), University Medical Center Groningen, University of Groningen, Groningen, The Netherlands.

**Keywords:** esophageal pressure, maximal inspiratory pressure, mechanical ventilation, respiratory insufficiency, respiratory mechanics

## Abstract

**OBJECTIVE:**

To compare the level of agreement between proximal (near the subject) and distal (inside the ventilator) measured airway occlusion pressure at 100 ms (P0.1) and occlusion pressure (Δ*P*_occ_), and to study the correlation between Δ*P*_occ_ and peak-to-trough esophageal pressure (Δ*P*_es_).

**DESIGN:**

Secondary analysis of prospectively collected physiology dataset (2021–2022).

**SETTING:**

Medical-surgical 20-bed PICU.

**PATIENTS:**

Children younger than 18 years with and without acute lung injury ventilated greater than 24 hours and spontaneously breathing with appropriate triggering of the ventilator.

**INTERVENTIONS:**

None.

**MEASUREMENTS AND MAIN RESULTS:**

Data from three expiratory hold maneuvers (with a maximum of three breaths during each maneuver) in 74 subjects (118 measurements) with median age 3 months (interquartile range 1–17), and primary respiratory failure due to a pulmonary infection in 41/74 (55.4%) were studied. The median proximal ∆*P*_occ_ was 6.7 cm H_2_O (3.1–10.7) and median P0.1 4.9 cm H_2_O (4.1–6.0) for the first breath from the maneuver; both increased significantly (*p* < 0.001) with the subsequent two breaths during the same maneuver. Median distal ∆*P*_occ_ was 6.8 (2.9–10.8) and P0.1 4.6 (3.9–5.6) cm H_2_O; both increased significantly (*p* < 0.001) with the two subsequent breaths. Proximal and distal Δ*P*_occ_ (*r* > 0.99, *p* < 0.001) and P0.1 (*r* > 0.80, *p* < 0.001) were correlated. Correlation between ventilator displayed and Y-piece measured Δ*P*_occ_ (*r* > 0.99) and P0.1 (*r* = 0.85) was good. Mean (sd) difference for Δ*P*_occ_ was 0.13 (0.21); levels of agreement were –0.28 and 0.54. For P0.1, mean (sd) difference was –0.36 (1.14) and levels of agreement –2.61 and 1.88. There was a high correlation between Δ*P*_es_ and ∆*P*_occ_ (*r* = 0.92) for the same breath and a good correlation with Δ*P*_es_ from the preceding breath (*r* = 0.76). There was a poor correlation with the transpulmonary pressure (*r* = 0.37).

**CONCLUSIONS:**

Δ*P*_occ_ is not affected by measurement site, whereas P0.1 may be overestimated or underestimated. Δ*P*_occ_ was highly correlated with the peak-to-trough esophageal pressure, supporting the concept that inspiratory effort can also be quantified noninvasively by measuring Δ*P*_occ_.

**RESEARCH IN CONTEXT**

- Excessive respiratory drive and inspiratory effort can cause patient self-inflicted lung injury.
- P0.1 estimates respiratory drive and peak-to-trough airway pressure during an expiratory hold (Δ*P*_occ_) quantifies inspiratory effort.
- It is unclear if measurement site (in the ventilator vs. at the Y-piece) influences P0.1 and Δ*P*_occ_.

**AT THE BEDSIDE**

- Measurement site is not affecting measured values of P0.1 and Δ*P*_occ_.
- Levels of agreement for P0.1 were considerable, indicating possible overestimated or underestimation.
- Δ*P*_occ_ has a good correlation with the peak-to-trough esophageal pressure during an expiratory hold and is therefore a good noninvasive estimate of inspiratory effort.

Allowing mechanically ventilated patients to breath spontaneously has several advantages, including improved ventilation distribution especially in dependent lung regions and improved ventilation-perfusion (1–3). Notwithstanding these beneficial effects, a number of studies report detrimental effects of spontaneous breathing when there is co-existing severe lung injury. Increased patient respiratory drive and effort (i.e., vigorous breathing) in response to abnormal gas exchange can amplify regional lung stress and strain, especially in lung dependent zones, causing or worsening lung injury (4–9). This is known as patient self-inflicted lung injury (P-SILI) and shares similar pathophysiological mechanisms to ventilator-induced lung injury including lung edema and pendelluft (5, 6). In addition, increased patient effort leads to diaphragmatic injury and fatigue as well as patient-ventilator asynchrony, also resulting in adverse patient outcome (7, 10, 11). Factors associated with a high respiratory drive include hypercarbia, acidemia, pain, anxiety, fever, and hypoxemia—all of which are common in the ICU (6).

Taken together, this pathophysiology underscores the importance of monitoring patient respiratory drive and effort to assess lung stress and diaphragmatic effort. If the brain-respiratory axis is intact, the strength of inspiratory effort is related to the respiratory drive (12, 13). The dynamic transpulmonary driving pressure (Δ*P*_L, dyn_) reflects the amplitude of regional lung stress (14). Esophageal pressure manometry is required to measure Δ*P*_L, dyn_, but this technique is challenging and not universally available on all ventilators (15). An alternative is the deflection in the airway pressure generated during the patient’s respiratory effort against an occluded airway (Δ*P*_occ_). Δ*P*_occ_ correlates with the pressure generated by the respiratory muscles to expand the lungs and chest wall during mechanically assisted breaths and may thus provide a noninvasive means of detecting patient effort and lung stress (16, 17). The respiratory drive cannot be measured directly, but the airway occlusion pressure at 100 ms (P0.1) has been proposed as a good indicator. P0.1 is a simple and noninvasive maneuver available on most modern ICU ventilators. It is not influenced by the patient’s (un)conscious reaction or respiratory mechanics due to the absence of airflow and insufflated volume during the maneuver (18, 19). In adults, P0.1 greater than 5 cm H_2_O is associated with increased respiratory muscle effort (12, 13, 20).

P0.1 and Δ*P*_occ_ are almost always measured inside the ventilator. Distally measured pressure correlates with pressures measured at the Y-piece of the patient circuit as long as there is zero-flow (21). However, P0.1 or Δ*P*_occ_ are measured under dynamic flow conditions as the patient is taking a breath. We are neither aware of definitive pediatric data and studies investigating the reliability of P0.1 and Δ*P*_occ_ measurements distal from the patient, nor how accurately the peak-to-trough esophageal pressure is reflected in Δ*P*_occ_ aside from one recent pediatric report (22). Understanding the accuracy of P0.1 or Δ*P*_occ_ readings is important as there is the need to establish pediatric normal values and how to differentiate between low, high inspiratory effort, or central drive. We therefore sought to study the level of agreement between P0.1 or Δ*P*_occ_ measured distal (i.e., measured in the ventilator) and measured at the Y-piece (i.e., proximal) in a heterogeneous cohort of mechanically ventilated children. We also studied the correlation between Δ*P*_occ_ and the peak-to-trough esophageal pressure.

## MATERIALS AND METHODS

This report is about a secondary analysis of prospectively collected physiology data (February 2021 to January 2022) from mechanically ventilated children (younger than 18 yr) with and without acute lung injury admitted to the 20-bed tertiary medical-surgical PICU of the Beatrix Children’s Hospital, University Medical Center Groningen (UMCG), Groningen, the Netherlands.

The original study underwent institutional review board (UMCG no. 2017.599) review and approval on November 1, 2017 (title “Airway occlusion pressures in mechanically ventilated children: a pilot study”), as outlined previously (23). The current secondary analysis did not require post hoc consent, and all work described here was carried out in accordance with the Declaration of Helsinki 1975 for research involving humans. The subjects in the data cohort had been mechanically ventilated for at least 24 hours and were triggering the ventilator. They received standard care using the institutional ventilator protocol described in the **Electronic Supplemental Materials** (http://links.lww.com/PCC/C597). Data from the following groups of subjects were excluded from the analyses, including those with any of the following conditions: neuromuscular disorder, premature birth with corrected gestational age younger than 40 weeks, traumatic brain injury (suspected), dysfunction of phrenic nerve or diaphragm, severe pulmonary hypertension, chronic lung diseases with mechanical home ventilation, managed with high frequency oscillation ventilation or with an endotracheal tube (ETT) leak greater than 18%.

### Data Acquisition

**Figure 1.**
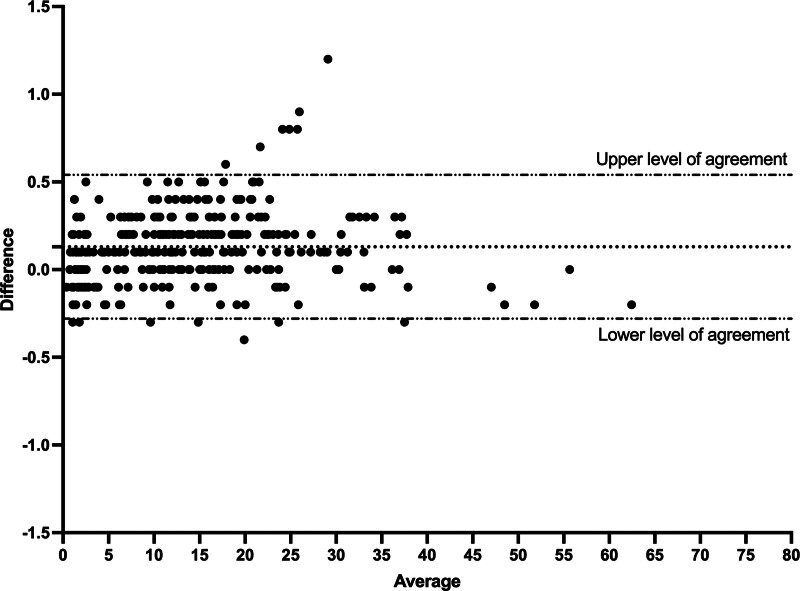
Bland Altman analysis of the difference between ventilator displayed and proximal measured airway occlusion pressure (Δ*P*_occ_). *Dotted line* represents the mean difference.

A pressure transducer was used to measure proximal airway pressure (*P*_aw PROX_) and a VarFlex flow sensor (Vyaire, Mettawa, IL) was placed at the Y-piece near the ETT and connected to the New Life Box (NLB) pulmonary function monitor (Applied Biosignals, Weener, Germany) (**Electronic Supplemental Materials**, **Fig. 1**, http://links.lww.com/PCC/C597). At the time of data acquisition, the subject had an esophageal catheter; we identified the optimal balloon volume as described elsewhere before the data acquisition and connected to the NLB (23). The airway pressure (*P*_aw_) measured by the AVEA ventilator (Vyaire Medical, Irvine, CA) (*P*_aw DISTAL_) was acquired via the analog output port using an analog to digital converter. All data were sampled at 200 Hz and subsequently offline analyzed using custom-built software (Polybench, Applied Biosignals, Weener, Germany). No other ventilator than the AVEA ventilator was used in this study.

Patient baseline characteristics including age, gender, weight, admission diagnosis, and medical history were used to characterize the study population. Ventilator settings had been recorded before the start of any study procedure and included ventilation mode, set positive end-expiratory pressure (PEEP), set pressure above PEEP, mean airway pressure (*P*_mean_), expiratory tidal volume (*V*_te_) normalized to actual bodyweight (*V*_te_/kg), set mandatory breath rate, inspiratory time, and Fio_2_. We also had access to the end tidal carbon dioxide, endotracheal tube (ETT) size, and Comfort Behavior Score as an estimate of patient comfort (24). All patients had a cuffed ETT in situ; adequate cuff pressure is assessed as least three times per day per a unit-specific nursing algorithm. In our practice, subjects were routinely instrumented with a 6 Fr (8 Fr in older children and adolescents) SmartCath esophageal catheter (Vyaire, Mettawa, IL). Balloon filling volume was individualized as previously reported in this study dataset (23).

### Study Procedure

Data had been collected on weekdays at 8:00 am upon availability of research staff. For measuring Δ*P*_occ_, we had performed at least three end-expiratory holds (separated by at least 60 s) using the MIP/P0.1 maneuver on the AVEA mechanical ventilator (Vyaire) because at least three maneuvers are needed to obtain a reliable P0.1 (25). The maneuver was performed per the manufacturer’s manual. Briefly, during the maneuver the negative deflection in the pressure tracing during the patient’s active effort to demand a breath is measured. The inspiratory flow valve remains closed so that no inspiratory flow is delivered (i.e., static conditions). The duration of the expiratory hold was 5 seconds in subjects less than 10 kg and 6 seconds in subjects greater than 10 kg (per the manufacturer’s manual), with the sensitivity (i.e., the level below PEEP that the airway pressure must drop, which determines the onset of a patient effort) set at 2 cm H_2_O.

### Outcomes

The primary outcome of this study was the difference between the proximally and distally measured Δ*P*_occ_ and P0.1. The secondary outcome was the correlation between Δ*P*_occ_ and peak-to-trough esophageal pressure (i.e., Δ*P*_es_).

### Data Analysis

**Figure 2.**
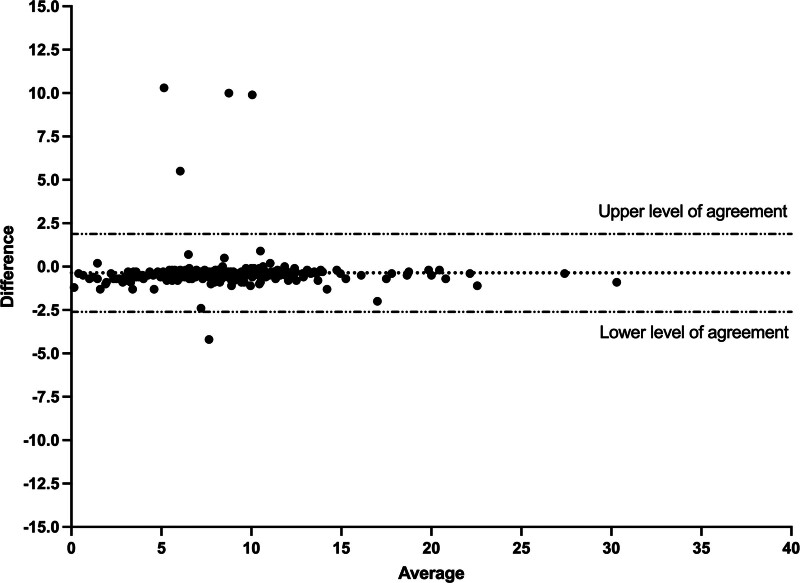
Bland Altman analysis of the difference between ventilator displayed and proximal measured P0.1. *Dotted line* represents the mean difference.

To calculate the Δ*P*_occ_ and P0.1 measured at the Y-piece, we manually identified the onset of inspiration in the proximal measured flow—time scalar as a positive increase in inspiratory flow (**Electronic Supplemental Materials**, **Fig. 2**, http://links.lww.com/PCC/C597). From this flow—time scalar, we calculated P0.1 from the decrease in *P*_aw_ from end-expiration during the first 100 ms after the onset of inspiration. The total drop in *P*_aw_ from PEEP during each maneuver was recorded as a measurement of occlusion pressure (Δ*P*_occ_). We also quantified the transpulmonary pressure (*P*_tp_) as marker of lung stress during the minute preceding the measurements (17). The three first breaths obtained during the maneuver were used (so each individual maneuver consists of the first three breaths) and three maneuvers represented one measurement (i.e., time of data collection). For the whole cohort, we then calculated the median MIP/P0.1 value. Subjects may have multiple measurements performed on different days of admission if eligible for inclusion.

### Statistics

Normality of data was assessed by the Kolmogorov-Smirnov test. Continuous data are presented as mean (± sd), when normally distributed, and as median (interquartile range [IQR]) for non-normally distributed data. Friedman tests were used to test the differences in ∆*P*_occ_ and P0.1 over time. For the primary and secondary outcomes, we calculated the Spearman correlation coefficient between the distally and proximally measured ∆*P*_occ_ and P0.1. We also used the Spearman correlation coefficient to analyze the correlation between distal ∆*P*_occ_ and the ∆*P*_es_ of the breath preceding the maneuver and *P*_tp_. Bland-Altman analyze were performed to estimate the levels of agreement. *p* values of less than 0.05 were accepted as significant. All statistical analyses were performed with SPSS 28 (IBM, Chicago, IL).

## RESULTS

We included data from 74 subjects (i.e., 118 measurements), with median age 3 months (IQR 1–17) and primary respiratory failure due to a pulmonary infection in 41 (55.4%) subjects (**Table 1**). Total ventilation time of the cohort was median 120 hours (IQR 96–216). Δ*P*_occ_ and P0.1 were measured for the first time after a median ventilation time of 96 hours (IQR 72–144) and 48 hours (IQR 24–120) before extubation. After exclusion of failed attempts or erroneous tracings, 18 of 95 (19.5%) measurements were available for analysis. In all, 43 of 74 (58%) subjects had Δ*P*_occ_ and P0.1 measured only once; 20 of the remaining 31 subjects had two consecutive measurements.

**TABLE 1.** Summary Data in 74 Patients Analyzed

Variable: Median (Interquartile Range) or *n* (%)	Value
Age (mo)	3.0 (1.0; 17.0)
Male *n* (%)	41 (55.4)
Weight (kg)	5.9 (4.0; 11.5)
Diagnosis (%)	
Pulmonary infection	55.4
Sepsis	1.4
Post-cardiac surgery	21.6
Post-non-cardiac surgery	8.1
Miscellaneous	12.2
Congenital heart defect	1.4
Endotracheal tube ≤ 5 mm (%)	93.2
Ventilator mode	
Pressure control/assist control	52.7
Continuous positive airway pressure + pressure support	45.9
Pressure above positive end-expiratory pressure (cm H_2_O)	12.0 (8.0; 14.0)
Positive end-expiratory pressure (cm H_2_O)	6.0 (5.0; 6.0)
Expiratory tidal volume normalized to bodyweight (mL/kg)	6.7 (5.9; 8.0)
Total breath rate (/min)	32 (25; 44)
End-tidal co_2_ (kPa)	6.6 (6.2; 7.1)
Fraction of inspired oxygen	0.30 (0.25; 0.35)
Transcutaneous measured oxygen saturation (%)	97 (96; 98)
Comfort B score	12 (11; 14)
Ventilation time before first measurement (hr)	96 (48; 120)
Ventilation time between first measurement and extubation (hr)	48 (24; 96)
Total ventilation time (hr)	120 (96; 216)

Description of the study population. Data are expressed as median (interquartile range) for continuous data and percentage of total for dichotomous data.

### Proximal vs. Distal Δ*P*_occ_ and P0.1

At each measurement, Δ*P*_occ_ increased significantly over the breaths and maneuvers. The median proximal ∆*P*_occ_ was 6.7 (3.1–10.7) cm H_2_O and median distal ∆*P*_occ_ was 6.8 (2.9–10.8) cm H_2_O, botch increased significantly (*p* < 0.001) with the subsequent two breaths during the same maneuver. For the whole cohort, the median proximal Δ*P*_occ_ for the first maneuver was 8.3 cm H_2_O (IQR 4.1–13.1). For the second and third maneuver this was 8.8 (4.6–12.8) and 9.1 cm H_2_O (4.2–13.4), respectively. Median distal Δ*P*_occ_ for the first maneuver was 8.5 cm H_2_O (IQR 4.0–13.2); for the second and third maneuvers this was 8.9 (4.1–13.0) and 9.1 (4.4–13.5) cm H_2_O, respectively. The proximal and distal Δ*P*_occ_ correlated significantly for all three maneuvers aggregated (*r* > 0.99, *p* < 0.001), but the difference between proximal and distal Δ*P*_occ_ was different (*p* < 0.001). Sensitivity analyses on each maneuver separately showed similar results.

At each measurement, P0.1 increased significantly over the breaths and not over the maneuvers. The median proximal P0.1 for the first breath from the maneuver was 4.9 cm H_2_O (4.1–6.0) and the distal P0.1 was 4.6 cm H_2_O (3.9–5.6). Both increased significantly (*p* < 0.001) with the subsequent two breaths during the same maneuver. The median proximal P0.1 for the first maneuver was 5.5 cm H_2_O (IQR 3.8–6.8). For the second and third maneuvers, this was 5.2 (4.1–6.6) and 5.4 (4.1–6.6) cm H_2_O, respectively. Median distal P0.1 for the first maneuver was 5.2 cm H_2_O (IQR 3.8–6.8); for the second and third maneuvers this was 4.9 (3.6–6.2) and 5.0 (3.8–6.0) cm H_2_O, respectively. The proximal and distal P0.1 correlated significantly for all three maneuvers aggregated (*r* > 0.80, *p* < 0.001), but the difference between proximal and distal P0.1 was significant (*p* < 0.001). Sensitivity analyses on each maneuver separately showed similar results.

### Correlation Between Ventilator Displayed and Y-Piece Measured Δ*P*_occ_ and P0.1

The correlation between ventilator displayed and Y-piece measured Δ*P*_occ_ was very high (*r* > 0.99; the mean (sd) difference was 0.13 cm H_2_O (0.21) and the lower and upper level of agreement were –0.28 and 0.54 cm H_2_O, respectively (**Fig. 1**). For P0.1, the correlation between ventilator displayed and Y-piece measured was also very good (*r* = 0.94; the mean (sd) difference was –0.36 cm H_2_O (1.14) and the lower and upper level of agreement were –2.61 and 1.88 cm H_2_O, respectively (**Fig. 2**).

### Correlation Between ∆*P*_occ_ and ∆*P*_es_

Thirty-three subjects (44.6%) had an esophageal catheter in situ, yielding 61 measurements. Of those, 51 of 61 (83.4%) measurements were eligible for data analysis after visual inspection of the esophageal pressure time scalar. In 24 of 51 (44.4%), the *∆P*_es_/*∆P*_aw_ ratio was between 0.7 and 1.3. In these measurements, there was a high correlation between ∆*P*_es_ and Δ*P*_occ_ for the same breath (*r* = 0.92); the correlation remained good when all ∆*P*_es_ tracings irrespective of ∆*P*_es_/*∆P*_aw_ ratio were analyzed (*r* = 0.75). There was a good correlation between ∆*P*_es_ from the breath preceding and Δ*P*_occ_ (*r* = 0.76); however, the correlation with the *P*_tp_ was only *r* equals to 0.37. Limiting the analysis to one data point, ∆*P*_es_ could be mathematically predicted by the equation 0.329 * Δ*P*_occ_, but the *R*^2^ was only 0.52.

## DISCUSSION

In this secondary analysis of curated, mechanical ventilation, physiologic data from our PICU in 2021–2022, we have shown that Δ*P*_occ_ as an estimate of patient inspiratory effort and P0.1 as an estimate of respiratory drive can be measured in children. Of note, Δ*P*_occ_ was not influenced by measurement site, but there were wide levels of agreement with P0.1. We identified good correlation between ventilator-displayed values of Δ*P*_occ_ and P0.1. We also found that P*o*_ck_ was highly correlated with peak-to-trough esophageal pressure, which supports the idea that patient inspiratory effort can be quantified noninvasively by measuring Δ*P*_occ_.

Vigorous breathing, especially in severe lung injury, may lead to P-SILI, underscoring the need for taking measures to assess inspiratory respiratory effort and drive. P0.1 has been proposed as an estimate of respiratory drive (13, 16, 26), and the gold standard to quantify respiratory effort is esophageal pressure manometry. However, the measurement of esophageal pressure that is not available on all mechanical ventilators, is invasive (which may be undesirable in children) and technically challenging because of catheter positioning, especially in small children. In contrast, Δ*P*_occ_ has been proposed as suitable alternative to quantifying respiratory effort (13, 16, 26). Some ventilators can automatically calculate Δ*P*_occ_, and most can calculate P0.1, but it is unclear how accurate these readings are in children. We know that airway pressure is best measured at the proximal airway, which may be important in young children, but most contemporary ventilators do not offer this possibility. However, mechanical ventilators can measure expiratory proximal pressure distal to the inspiratory valve during the expiratory phase but the accuracy of this measurement is unclear, especially in young children. This limitation may affect the value of Δ*P*_occ_ and P0.1. Even so, our findings in one commercially available ventilator showed very high correlation between distal and proximal measurements.

A post hoc analysis of three clinical studies by Telias et al (13) comparing different ventilators and a clinical study found that the values displayed by mechanical ventilators accurately reflected P0.1. Importantly, from this investigation, the authors concluded that P0.1 may be underestimated by the ventilator when it is calculated without performing a hold (i.e., under dynamic flow conditions). This phenomenon has also been reported by others (27). In our study, we identified a strong correlation between P0.1 displayed by the ventilator and P0.1 measured at the Y-piece, but the level of agreement analysis indicated that actual P0.1 might be overestimated or underestimated. This result may in part be explained by the lack of acknowledgement as to where to position the markers in the airway tracing that identify the onset of the first 100 ms. Ventilators have built this estimate built into the proprietary algorithm. We manually set the markers in the airway tracings from the Y-piece, and we cannot rule out that the possibility this our marker setting differed from the one incorporated in the ventilator algorithm. Also, the AVEA ventilator requires setting a sensitivity *P*_aw_ level to detect an inspiratory effort; at present it is unclear if and how this sensitivity level should be set in young children.

In adults, thresholds values of P0.1 of excessive (3.5–4.0 cm H_2_O) and low inspiratory effort (1.1 cm H_2_O) have been proposed. Recently, Ito et al (22) reported similar numbers from a secondary analysis of an ongoing randomized trial testing a lung and diaphragm protective ventilation strategy in children. Our analysis was not designed to identify threshold values, and further studies are needed to examine threshold values in children.

In our study, we examined the correlation between Δ*P*_occ_ and Δ*P*_es_ in a subgroup of children. There was good correlation between Δ*P*_es_ of the breath preceding the breath in which Δ*P*_occ_ was measured, indicating that if Pes manometry is unavailable Δ*P*_occ_ is a suitable alternative. Similar findings were reported by Ito et al (22). In adults, even though Δ*P*_occ_ correlates fairly well with lung stress, we failed to confirm this correlation in our study—but we were only able to analyze a 1-minute period, which may not have been representative for the actual lung stress (17). Furthermore, alternative explanations for the observed differences may include small sample size and the fact that disease severity in our cohort was relatively mild with inherently low *P*_tp_.

The findings in our study, together with previous observations, support the idea that bedside measurement of Δ*P*_occ_ and P0.1 can be used to quantify respiratory effort and drive. However, the length of the expiratory hold may potentially influence the values obtained, and thus further study is required. Also, as we found that values for Δ*P*_occ_ and P0.1 significantly increased over the number of attempts, clinicians should not rely on just one measurement.

There are some limitations to our study that need to be discussed. First, our study was not designed to address differences in ventilator performance when measuring Δ*P*_occ_ and P0.1, as we only use one ventilator type on our PICU. We recognize that the AVEA comes with a MIP/P0.1 option, which is not the case for other commercially available ventilators. In addition, our study was a single-center study including mainly young children. Although in our opinion the study population is representative of the PICU population in general, both limitations may limit generalizability of our findings and warrant validation in other cohorts. Second, our study was designed as a physiologic study, thus the clinical usefulness of Δ*P*_occ_ and P0.1 measurement in regard to patient outcome needs further evaluation. Our study included a convenience sample and was designed to examine whether measurement site affected the readings in children.

In conclusion, in this secondary analysis of physiologic data we have found that Δ*P*_occ_ can be measured in mechanically ventilated children and is not affected by measurement site. Levels of agreement for P0.1 were wide, indicating overestimated or underestimating the true value of P0.1 compared with what the ventilator displays. Δ*P*_occ_ was highly correlated with the peak-to-trough esophageal pressure, supporting the concept that patient respiratory effort can also be quantified noninvasively by measuring Δ*P*_occ_.

## ACKNOWLEDGMENTS

The authors thank Jasmijn Teunissen and Douwe van der Steen for their participation in the data collection.

## Supplementary Material
