# Supplementary material for: Airway Occlusion Pressure and P0.1 to Estimate Inspiratory Effort and Respiratory Drive in Ventilated Children
Source: Pediatr Crit Care Med. 2025 Feb 13;26(4):e498–506. doi: 10.1097/PCC.0000000000003697 (PMC11960681; doi:10.1097/PCC.0000000000003697)

Electronic Supplemental Material

Airway occlusion pressure and P0.1 to estimate inspiratory effort and respiratory drive in ventilated children

Michelle W. Rudolph, MD; m.w.rudolph@umcg.nl (1)

Maaike Sietses, MD; m.sietses@umcg.nl (1)

Alette A. Koopman, MSc; a.a.koopman@umcg.nl (1)

Robert G.T. Blokpoel, MD; r.g.t.blokpoel@umcg.nl (1)

Martin C.J. Kneyber, MD PhD FCCM; m.c.j.kneyber@umcg.nl (1,2)

(1) Department of Paediatrics, Division of Paediatric Critical Care Medicine, Beatrix Children's Hospital, University Medical Center Groningen, University of Groningen, Groningen, the Netherlands; (2) Critical care, Anaesthesiology, Peri-operative & Emergency medicine (CAPE), University Medical Center Groningen, University of Groningen, Groningen, the Netherlands

Ventilator protocol

Patients were intubated with a cuffed ETT (KimVent, Microcuff Endotracheal Tube, Paediatrics, Roswell, USA). A neonatal breathing circuit (diameter 10 mm) is used when ventilating patients < 10 kg; other, we use the 22 mm diameter smoothbore breathing circuit (Intersurgical LTD, Berkshire, UK)

We exclusively ventilate patients in a PC ventilation mode (AVEA^{TM}, Vyaire, Mettawa, Ill, USA), limiting inspiratory pressures £ 28 cmH_2O (£ 32 cmH_2O when there was clinically suspected decreased chest wall compliance) and expiratory Vt (Vt-exp) 5 – 7 mL/kg actual bodyweight (as there was no obesity in the patient cohort [i.e., no patient with SDS < -2 SD or > +1 SD]). Vt-exp was measured near the Y-piece in children < 10 kg using a proximal flow sensor (VarFlex^{TM}, Vyaire, Mettawa, Ill, USA). We only use cuffed endotracheal tubes(ETT). A heat moisture exchanger (Gibeck, Teleflex Medical, Vianen, The Netherlands) was in situ between the patient circuit and the ETT.

Setting the ventilator breath rate is dictated by the underlying pathology and age; we routinely carefully monitor the flow-time scalar to identify if the inspiratory time setting was appropriate and there was no development of intrinsic PEEP. This means that in our unit the I:E ratio is not fixed. Initial PEEP is 4-6 cm H_2O and further titrated at the discretion of the attending physician, targeting SpO_2 88 – 92% for patients with lung injury. Unless dictated otherwise, target pH is > 7.20. Patients who breathed spontaneously were not studied as the AVEA does not allow for Pplat to rise above PIP during an inspiratory hold. High-frequency oscillatory ventilation (HFOV) is used

per unit-specific algorithm as described elsewhere in patients who cannot be supported within the aforementioned conventional ventilation targets. Use of neuromuscular blocking agents was at the discretion of the bedside team.

Figure 1 Cartoon explaining the set-up

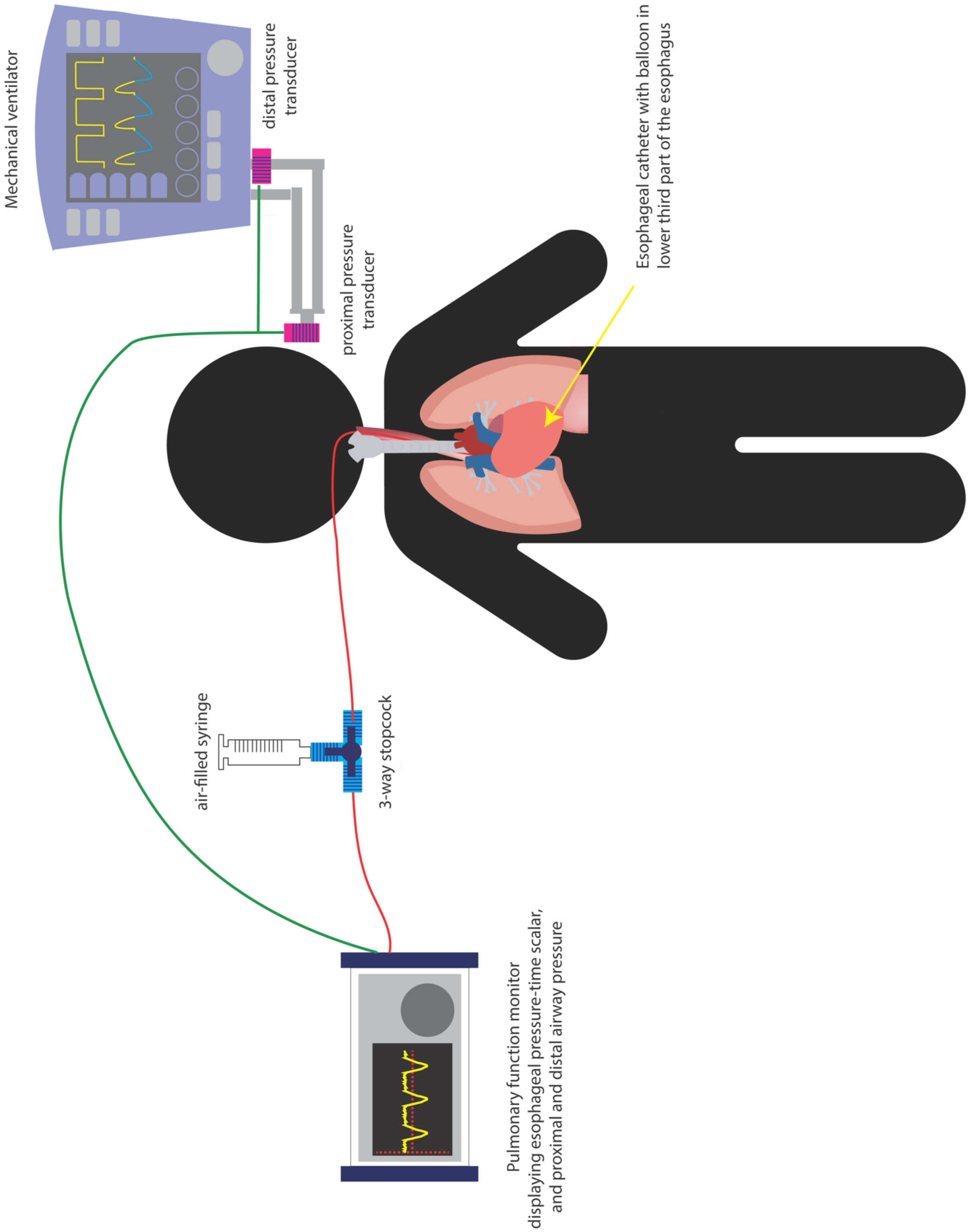

Figure 2 Explanation of waveform analysis

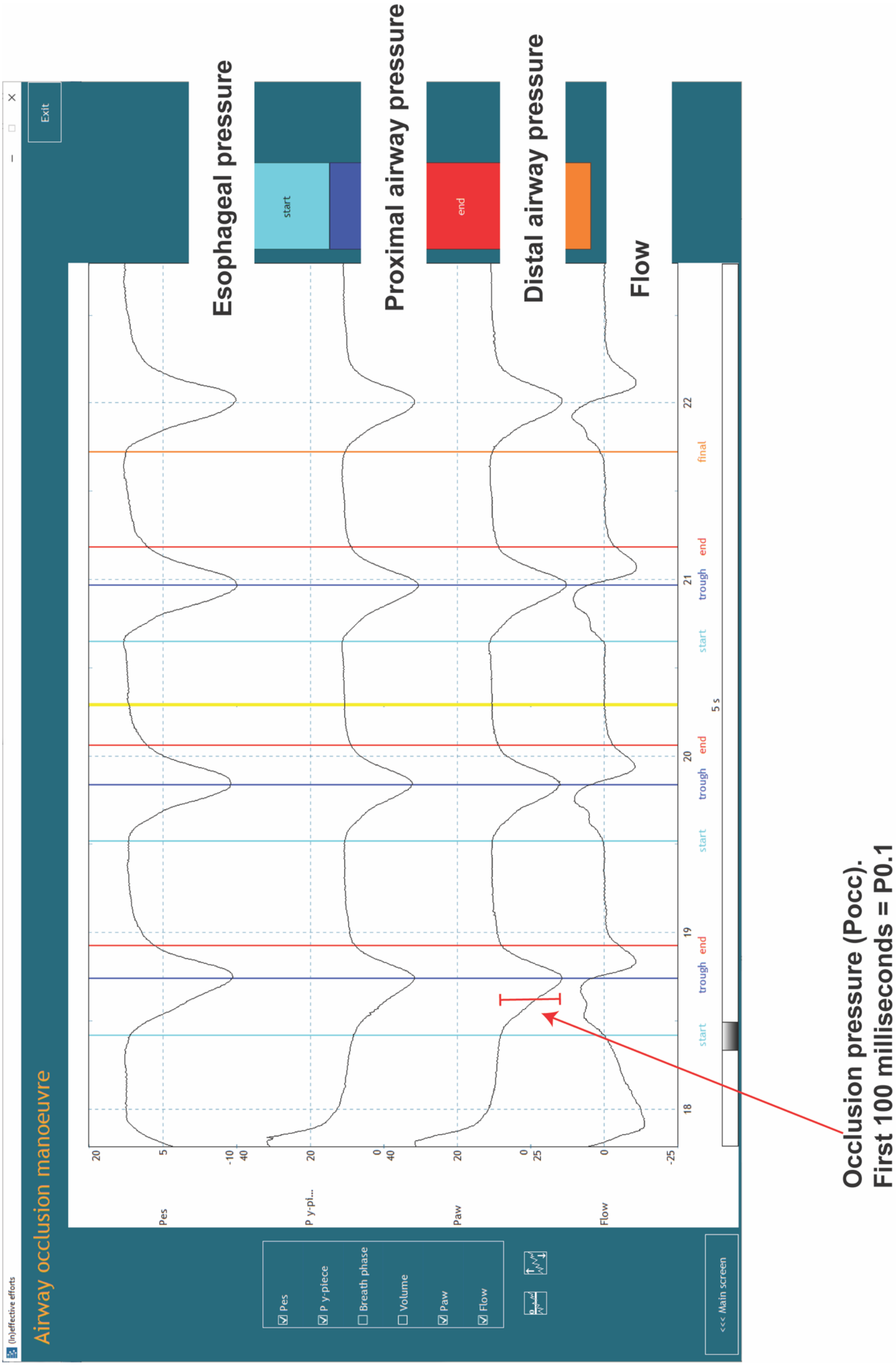

Supplement: Supplementary file 1 [file pcc-26-e498-s001.pdf]
